# The new gold rush in clinical diagnostics: from standard laboratory assays to fast and accurate point-of-care (POC) methods

**DOI:** 10.1128/spectrum.01887-25

**Published:** 2025-09-19

**Authors:** Hui-Chen Foreman

**Affiliations:** 1 Mercy LLC, Roswell, Georgia, USA; City of Hope Department of Pathology, Duarte, California, USA

**Keywords:** INAA, MCDA, LFA, clinical diagnosis, isothermal nucleic acid amplification, qPCR, HBV, HCV, point of care, POC, hepatitis, hepatocellular carcinoma

## Abstract

Integrating two point-of-care (POC)-amenable technologies, namely, multiple cross displacement amplification (MCDA) and gold nanoparticle-based lateral flow biosensors (AuNPs-LFB), has demonstrated excellent detection of hepatitis B virus (HBV) and hepatitis C virus (HCV). In a recent study, Zhang et al. (H. Zang, Y. Shi, Z. Wu, Q. Zhao, et al., Microbiol Spectr 13:e01738-24, 2025, https://doi.org/10.1128/spectrum.01738-24) developed a novel, single-tube, multiplex MCDA-AuNPs-LFB assay that targets the HBV S gene and the HCV 5′-UTR region. The assay operates under isothermal conditions at 64°C for 35 min, and the AuNPs-LFB assay permits a visual interpretation of results. This platform achieves an analytical sensitivity comparable to lab-based quantitative PCR but with shorter turnaround time, reduced cost, operational simplicity, and undetectable cross-reactivity to anatomically relevant, non-target pathogens, making it highly suitable for decentralized POC diagnostic applications. The success of hybrid systems in simplifying nucleic acid testing while increasing portability indicates a potential transition in clinical diagnosis from centralized tech-centered labs to local clinics, field testing, and even home care.

## COMMENTARY

Molecular diagnostics play a pivotal role in identifying pathogens that drive infectious diseases, directly influencing patient management and public health interventions. By targeting pathogen-specific nucleic acid sequences, molecular diagnostics enable accurate detection at the genus or species level—and in some cases, allow for the identification of single-nucleotide polymorphisms (1). Among these, PCR-based methods remain essential clinical tools in the epidemiological surveillance for and control of emerging pathogens and drug-resistant strains, offering high sensitivity, specificity, and rapid turnaround (2).

Clinical Laboratory Improvement Amendments- and Food and Drug Administration-compliant laboratories commonly adopt quantitative PCR (qPCR) due to its straightforward primer design, assay optimization, and implementation. Its high analytical sensitivity, specificity, reproducibility, and 10–100-fold speed advantage over traditional culture methods (3) positions qPCR as the gold standard. However, high-burden infectious diseases, such as hepatitis B and C (HBV/HCV), *Mycobacterium tuberculosis*, and *Plasmodium falciparum*, disproportionately affect low-resource settings that lack access to advanced instrumentation and trained personnel. Therefore, a challenge remains in developing molecular diagnostics that retain qPCR’s advantages while offering cost-effective, rapid results, and operational simplicity more suited for decentralized or point-of-care (POC) use.

Isothermal nucleic acid amplification (INAA) presents a cost-effective and practical alternative to qPCR for nucleic acid detection, particularly in point-of-care settings (4). INAA enables amplification at a constant temperature, eliminating the need for thermocyclers and aligning well with the World Health Organization’s ASSURED criteria for diagnostics in resource-limited environments (5). However, the lack of thermal cycling may raise concerns regarding assay specificity due to reduced stringency in primer annealing. For instance, loop-mediated isothermal amplification (LAMP), the most widely accepted INAA platform, requires careful empirical testing for its primer pairs (four–six primers) to achieve high specificity. Wang et al. developed an isothermal amplification method, called the multiple cross displacement amplification (MCDA), in 2015 (6). Based on the strand displacement principle, MCDA utilizes a set of 10 primers targeting distinct regions within a specific gene, enhancing assay specificity and sensitivity to that of qPCR (7). Notably, MCDA achieves faster amplification kinetics than LAMP or a qPCR method (8).

Recently, Zhang et al. (9) developed an integrated point-of-care (POC) diagnostic platform by combining a molecular-based approach (MCDA-INAA) with an antibody-based gold nanoparticle lateral flow assay (LFA) to enable rapid, visual detection of HBV and HCV. HBV and HCV infections represent a significant global public health burden, contributing substantially to acute and chronic liver diseases, including hepatocellular carcinoma (10). Initiating timely treatment and curbing transmission require accurate, early diagnosis.

In this proof-of-concept study, the authors designed MCDA primers targeting conserved regions of prevalent HBV and HCV genotypes and subtypes circulating in China. For HBV, a conserved sequence within the HBV surface antigen (HBV-S) was selected to encompass genotypes B, C, and D and recombinant forms B/C and C/D. For HCV, the conserved 5′ untranslated region (5′-UTR) was chosen to detect subtypes 1b, 2a, 3a, 3b, and 6a. The MCDA reaction was conducted in a single-tube, multiplex format using Bst 2.0 polymerase and AMV reverse transcriptase (for RNA templates), which facilitates strand displacement for both DNA (HBV) and RNA (HCV) targets.

To distinguish the two viral targets, dual-labeled primers were employed: (i) FAM and biotin for HBV-MCDA amplicons and (ii) digoxigenin and biotin for HCV-MCDA amplicons. A gold nanoparticle-based lateral flow biosensor (AuNPs-LFB) provides a rapid, instrument-free antibody-based visual readout. The test strip comprises sequential pads facilitating capillary-driven fluid flow through three functional zones: (i) sample loading, (ii) streptavidin-conjugated gold nanoparticle capture of biotin-tagged amplicons, and (iii) immobilized antibodies specific for FAM and digoxigenin at test lines TL1 and TL2, respectively. TL1 positivity indicates HBV detection, while TL2 positivity indicates HCV detection. A control line (CL) coated with biotin captures excess streptavidin to confirm proper flow and test validity.

Plasmids encoding HBV-S and HCV-5′-UTR sequences were used to optimize the MCDA assay conditions. The reaction, performed isothermally at 64°C for 35 min, achieved a limit of detection (LoD) of 10 copies for either target, matching the sensitivity of standard qPCR and surpassing LAMP- and RPA-based HBV/HCV detection platforms. Importantly, no cross-reactivity was observed with other clinically relevant pathogens, including HAV, HIV, HSV, and other viral, bacterial, or fungal agents. A total of 107 serum samples from patients with suspected HBV and/or HCV infections at the Second Affiliated Hospital of Guizhou University of Traditional Chinese Medicine (May 2023–February 2024) provided clinical validation. Nucleic acids extracted using a commercial DNA/RNA purification kit were subjected to both MCDA and qPCR assays in parallel. The multiplex HBV/HCV-MCDA-AuNPs-LFB assay demonstrated 100% sensitivity and 100% specificity, fully concordant with the qPCR results, underscoring its potential utility in clinical diagnostics.

While the full clinical validation of this new IVD device—including assessments of interference substances and reagent/shipping stability—remains pending, several key attributes already highlight its potential. In this preliminary study, the HBV/HCV-MCDA-AuNPs-LFB assay demonstrated diagnostic accuracy equivalent to that of laboratory-based qPCR with reduced turnaround time and lower cost. The MCDA reaction was completed in approximately 35 min—faster than even many other isothermal amplification methods, such as LAMP. Including nucleic acid extraction, the total workflow was completed in under 50 min, significantly faster than standard qPCR protocols. Additionally, the device’s design supports use in low-resource environments by eliminating costly thermocyclers, fluorescence/turbidity readers, and specialized operator training. These features make the device especially attractive for decentralized settings, such as point-of-care clinics, field testing, and potentially even home-based diagnostics if combined with microfluidic or other nucleic acid extraction methods.

Given that the INAA method can achieve diagnostic accuracy comparable to conventional qPCR, but with significantly reduced turnaround time and cost, the following question arises: what is the continued necessity for qPCR? The advancement of INAA technologies is reshaping the clinical diagnostics landscape and is poised to become the next gold-standard technique. Importantly, this paradigm shift entails more than a technical enhancement; it necessitates a comprehensive evaluation for integration within existing healthcare frameworks, encompassing regulatory oversight, clinical workflow adaptation, and public health policy to ensure sustainable implementation.
